# Opportunities and Challenges of Prognostic Models for Extremely Preterm Infants

**DOI:** 10.3390/children10101712

**Published:** 2023-10-21

**Authors:** Angret de Boer, Pauline E. van Beek, Peter Andriessen, Floris Groenendaal, Marije Hogeveen, Julia S. Meijer, Sylvia A. Obermann-Borst, Wes Onland, Liesbeth (H. C. J.) Scheepers, Marijn J. Vermeulen, E. J. T. (Joanne) Verweij, Lien De Proost, Rosa Geurtzen

**Affiliations:** 1 Department of Neonatology, Amalia Children’s Hospital, Radboud University Medical Center, Geert Grooteplein Zuid 32, 6525 GA Nijmegen, The Netherlands; pauline.vanbeek@radboudumc.nl (P.E.v.B.); marije.hogeveen@radboudumc.nl (M.H.); rosa.geurtzen@radboudumc.nl (R.G.); 2 Department of Obstetrics and Gynecology, Leiden University Medical Centre, 2333 ZA Leiden, The Netherlands; e.j.t.verweij@lumc.nl; 3 Department of Neonatology, Máxima Medical Center, 5504 DB Veldhoven, The Netherlands; p.andriessen@mmc.nl (P.A.); julia.meijer@mmc.nl (J.S.M.); 4 Department of Neonatology, Wilhelmina Children’s Hospital, University Medical Center Utrecht, 3584 EA Utrecht, The Netherlands; f.groenendaal@umcutrecht.nl; 5 Care4Neo, Dutch Neonatal Patient and Parent Advocacy Organization, 3068 JN Rotterdam, The Netherlands; wetenschap@care4neo.nl (S.A.O.-B.); m.j.vermeulen@erasmusmc.nl (M.J.V.); 6 Department of Neonatology, Emma Children’s Hospital, Amsterdam University Medical Center, 1105 AZ Amsterdam, The Netherlands; w.onland@amsterdamumc.nl; 7 Amsterdam Reproduction & Development, 1105 AZ Amsterdam, The Netherlands; 8 Department of Obstetrics and Gynecology, Maastricht University Medical Centre, 6229 HX Maastricht, The Netherlands; hcj.scheepers@mumc.nl; 9 Department of Neonatal and Pediatric Intensive Care, Division of Neonatology, Sophia Children’s Hospital, Erasmus Medical Center, 3015 CN Rotterdam, The Netherlands; 10 Department of Ethics and Law, Leiden University Medical Centre, 2333 ZA Leiden, The Netherlands; l.de_proost@lumc.nl

**Keywords:** extremely preterm infant, decision making, prognostic models

## Abstract

Predicting the short- and long-term outcomes of extremely preterm infants remains a challenge. Multivariable prognostic models might be valuable tools for clinicians, parents, and policymakers for providing accurate outcome estimates. In this perspective, we discuss the opportunities and challenges of using prognostic models in extremely preterm infants at population and individual levels. At a population level, these models could support the development of guidelines for decisions about treatment limits and may support policy processes such as benchmarking and resource allocation. At an individual level, these models may enhance prenatal counselling conversations by considering multiple variables and improving transparency about expected outcomes. Furthermore, they may improve consistency in projections shared with parents. For the development of prognostic models, we discuss important considerations such as predictor and outcome measure selection, clinical impact assessment, and generalizability. Lastly, future recommendations for developing and using prognostic models are suggested. Importantly, the purpose of a prognostic model should be clearly defined, and integrating these models into prenatal counselling requires thoughtful consideration.

## 1. Introduction

Extremely preterm birth, defined as a gestational age below 28 weeks [1], is one of the main causes of perinatal morbidity and mortality [2]. With advancements in perinatal care and medical technology, the survival rates of extremely preterm infants (EPIs) have significantly improved in recent decades [3]. Nonetheless, improved survival rates raise the concern about adverse long-term outcomes in the increasing number of surviving EPIs [4]. Extremely preterm birth is associated with a variety of serious complications, including bronchopulmonary dysplasia, serious brain injury, and severe retinopathy of prematurity [5]. Predicting these short- and long-term outcomes for EPIs remains a complex challenge [6]. Accurate outcome estimations for EPIs play a pivotal role in counselling parents and planning a care trajectory. Multivariable prognostic models might be valuable tools, since individual patient characteristics can be included for the risk assessment stratification of adverse outcomes in EPIs [7,8,9,10].

Over the years, multiple prognostic models predicting outcomes after extremely preterm birth have been developed [7,8]. A recent systematic review showed that in recent decades, 144 models with varying quality have been developed for predicting mortality in EPIs [7]. However, the actual use of prognostic models in clinical practice is scarce and limited to online available calculators such as the NICHD (The Neonatal Institute of Child Health and Human Development) or NIC-PREDICT calculators [6,7].

Ongoing debates persist both within the Netherlands, where the authors are based, and within the international scientific, lay, patient, and parental communities. These discussions focus on various aspects such as the necessity for guidelines in the context of extreme prematurity, the requirements for personalized medicine, the utility/practicality of statistics, and the determination of meaningful outcomes for EPIs. There is growing emphasis on considering what outcomes matter most to parents, and it has been observed that the development of prognostic models providing a percentage likelihood of survival may not necessarily aid parents in decision making [11,12].

This motivated our multidisciplinary group of authors, who possess expertise in prediction models, counseling, decision making, and ethics and who also include parent representatives, to convene and deliberate on the development and utilization of prognostic models in the context of EPIs. The aim of this perspective is to elaborate on the use of prenatal prognostic models in EPIs by thoroughly exploring their challenges and opportunities described in the existing body of literature. Hereby, we hope to contribute to the careful development and use of these models in the future. In the first part of this paper, the use of prognostic models at a population level, and its opportunities and challenges, in the context of guideline development and policy making will be discussed. In the second part of this paper, the focus is shifted towards the opportunities and challenges raised by using prognostic models on an individual level for personalized counselling and informed decision making. The third section of this paper redirects attention towards further optimizing the development and application of prognostic models in the context of extreme prematurity. General statistical and methodological requirements, however, are beyond the scope of this paper. Lastly, we discuss future recommendations for the use of prognostic models in extreme prematurity.

## 2. Using Prognostic Models at a Population Level

Prognostic models may be helpful at a population level, for example, for the development of perinatal guidelines or for effective policy making to provide tools for benchmarking and allocating resources.

### 2.1. Perinatal Guidelines

The availability of prognostic models would be useful in developing perinatal guidelines if cut-off points reflecting either acceptable or unacceptable rates of adverse outcomes could be defined, considering the existence of a shared decision-making zone with parents in which both palliative comfort care and early intensive care are permissible, and recognizing the necessity for defining cut-off points within this zone. In the literature, calculated prognosis-based guidelines have been suggested as an alternative to gestational age-based guidelines for making decisions about providing neonatal care, such as decisions about resuscitation versus palliative comfort care [13,14,15]. However, especially when allowing for parental involvement in decision making, as is common in several high-income countries, the implementation of such prognosis-based guidelines requires defining clear cut-off points to define a grey zone [16,17]. In defining such points, two important ethical considerations arise: (I) How good or how bad does the prognosis of an infant born at the limit of viability have to be before the treatment options regarding life-sustaining treatment are altered? (II) What is the definition of a poor outcome justifying restricting treatment options [16]? Answering these questions and defining these cut-off points have been attempted, but turned out to be very difficult. In 2009, the American advisory committee of the Fetus and Newborn discussed a clinical report on delivery room care for infants born at an extremely low gestational age. In this clinical report, general guidelines on whether resuscitation should or should not be offered were suggested [16]. The members of the committee could not agree on precise morbidity and mortality thresholds for deciding when resuscitation would be an acceptable option, nor on a definition of ‘adverse outcomes’ [14,16]. In Canada, a guideline was developed that was initially based upon mortality and major neurodevelopmental disability risks as cut-offs for initiating intensive care treatment or palliative comfort care [17], but exact percentages were omitted in the final guideline [18]. Apparently, healthcare providers find it easier to establish treatment thresholds based on gestational age rather than the precise chance of a “good” or “adverse” outcome, even though gestational age could also be ‘translated’ to such a percentage—a conclusion also drawn from several surveys [14,19]. In conclusion, prognostic models could be of use in the development of perinatal guidelines for impending extremely preterm delivery, but their implementation may be complex. It is important to acknowledge that in certain countries, there may not be a grey zone and a universally proactive approach is consistently adopted [20,21,22,23]. In such contexts the application of prognostic models to facilitate guideline development with specific cut-off points may not be applicable/relevant. Nonetheless, prognostic models can still offer valuable insights and information to both physicians and parents. It is relevant that healthcare providers in the same country and the same quality of level III NICUs provide similar information to pregnant women and their partners, particularly when these pregnant women are transferred from one level III hospital to another one when obstetric high care beds or NICU beds are not available at that time. The development of a prognostic model in a particular country could be very helpful in decision making.

### 2.2. Policy Development

Prognostic models may support policy development in perinatal care, for example, by supporting resource allocation and benchmarking. Regarding benchmarking, interest in assessing quantitative comparisons of performance between Neonatal Intensive Care Units (NICUs) from different hospitals is growing. To ensure adequate comparisons, performance indicators should be adjusted for NICU patients’ characteristics, a process commonly referred to as “case-mix adjustment”. For this purpose, a patient-based prognostic model may be used, which describes in probabilistic terms the patient’s outcome as a function of the selected patient characteristics. This prognostic model may be applied to the patients of each NICU, leading to a predicted probability of outcomes. Finally, the ratio between observed and predicted outcomes can be calculated and may serve as a performance indicator to compare NICU outcomes [24].

Furthermore, prognostic models may be useful tools to help hospitals and healthcare systems to allocate resources efficiently [25,26,27,28]. By predicting the likelihood of, e.g., the length of hospital stay and adverse outcomes, healthcare facilities can plan ahead for the necessary beds and equipment, and for follow-up care that may be required to meet the specific needs of EPIs [25,26,27,28].

## 3. Using Prognostic Models at an Individual Level

Prognostic models are typically developed to estimate the chances of future outcome measures in individual patients. By accumulating multiple relevant variables and allowing for representation of the relative importance of each factor in predicting the final outcome, models may add to the transparency and precision of prognostic predictions in counselling [15].

### Individualizing Prenatal Counselling and Decision Making

Prognostic models may be helpful tools during prenatal counselling and decision making, especially in those countries and/or hospitals employing a “grey zone” in which shared decision making with parents is essential for the decision to initiate resuscitation or defer to palliative comfort care [22,29].

First, they enable the use of a combination of multiple factors to predict the risk of future adverse clinical outcomes in EPIs. Considering multiple factors in addition to gestational age might enable a more personalized risk assessment of adverse outcome [15]. It could help healthcare professionals and parents to better understand how these predictors interact and affect the prognosis. Currently, as far as we know, no guideline based on a prognostic model exists. However, recent guidelines for perinatal care at <26 weeks of gestation do try to incorporate multiple factors in addition to gestational age, such as the British Association of Perinatal Medicine guideline drawing on data from the UK and abroad [30,31]. Based on this guideline, an infographic was created to visualize the risk of a poor outcome across multiple factors, stratified into three groups: extremely high risk, high risk, and moderate risk of either dying or surviving with severe impairment. However, a prognostic model including a weight for each predictor and generating an exact outcome estimate is not provided [31]. Determining the influence of each factor on a specific outcome turned out to be challenging, as shown in a first evaluation of this guideline in practice, and variation among healthcare professionals may occur, especially where combinations of factors co-exist (e.g., gestational age 25 weeks, male sex, singleton) [30]. It may seem as if each of these factors carries equal weight, while in reality, the predictive value of certain factors may be greater than others. The use of a (multivariable) prognostic model accounts for this.

Second, using models may increase uniformity in outcome predictions during counselling. Research shows that neonatologists’ outcome predictions vary significantly within similar cases, possibly due to different interpretation of data, limited precision in prognostication, and varying attitudes regarding disability [32,33,34,35]. Another study shows that both obstetricians and pediatricians underestimate survival and survival without any comorbidities/disabilities in EPIs [34]. It is hypothesized that prognostic models may provide a more consistent approach among healthcare professionals towards pre-natal decision making, but no studies have been performed to support this hypothesis. Whether prognostic models can provide more precise and accurate prognostication compared to clinicians is unknown. It may be expected that prediction models provide smaller confidence intervals compared to a clinician’s uncertainty, but research confirming this expectation is lacking. Contrastingly, the use of prognostic models might promote a level of confidence that is unjustified given the limitations of prognostic modeling. These uncertainties inherent to prognostic estimates with its confidence intervals should be effectively communicated to parents [36,37,38,39].

However, even with better individualized prognosis estimations on, for example, survival, its relevance in shared decision making regarding resuscitation might be limited. General information on survival in prenatal counseling is important, and the ‘wish for survival’ for many parents is an important consideration in treatment decisions [40,41,42,43]. However, the literature suggests that parental decisions on resuscitation are not significantly affected by physicians’ exact predictions of survival or disability. Two randomized controlled trials showed no difference in treatment choices using a 30% versus a 60% chance of survival [44,45]. Another qualitative study interviewing experienced parents found that the prediction of survival was not central to parental decision making regarding resuscitation at the limit of viability [39]. The complexity of medical information, overwhelming emotions, and ongoing medical crises may contribute to the limited impact of physicians’ predictions on parental decision making [39,41,42,46,47]. Instead, parents are mainly influenced by their own perception of the possibilities of survival or may rely on instinct [40,41,42,45,48]. Nevertheless, the use of statistics serves other purposes beyond immediate decision making during counselling, such as providing insight and transparency, and anticipating the future [42].

In addition to survival, neurodevelopmental impairment (NDI) is often used as an outcome parameter in studies presenting the long-term outcomes of EPIs. This composite outcome measure includes cognitive scores, neurological functioning, vision, and hearing, but these specific outcome measures in themselves may hold little significance for parents; what matters to them is the translation of these measures into practical implications for their children and families [49,50].

Next to the discussion on the use of outcome parameters, it matters how these are communicated to parents. For example, adults who were born preterm expressed concerns that using a calculator- or model-derived prognosis could make people feel as if they are just a number [51]. Furthermore, the manner in which calculated risks and chances are communicated to parents matters [52]. For example, parents may consider quality of life important when making resuscitation decisions, but the interpretation differs among parents, e.g., ‘being without pain’, or ‘being able to play soccer’ [53]. Professionals must try to translate the outcome parameters used in a model (e.g., the risk on cerebral palsy) to the parental concerns (e.g., being able to play soccer).

Finally, the implementation of prognostic models may pose potential risks to the decision-making process. When the meaning of the outcome of a prognostic model is poorly understood, healthcare providers may feel that uncertainty in prognosis/prediction is reduced. This may inadvertently lead to more directive communication and diminish parental involvement in the decision-making process [54,55]. The opportunities and challenges described, are summarized in Table 1.

## 4. Considerations While Developing Prognostic Models for Extremely Preterm Infants

In the following paragraphs, we focus on a number of important considerations while developing prognostic models for EPIs.

First, it is complex to determine which factors to include as predictors in the prognostic model and how to define them. All predictors included in a prognostic model must have a contribution to the prediction and should be measurable, and the necessity of each individual factor should be carefully evaluated. Sufficient face validity may increase a user’s confidence in the model, and thus, their willingness to use the model in clinical practice. Face validity indicates to what extent a model gives the impression of measuring something relevant. For models concerning extreme prematurity, this means it might be difficult to convince a healthcare professional of the usefulness of a model without the predictor ‘gestational age’. However, the major function of a prediction model is optimizing the predictive accuracy of all combined factors together, rather than dissecting the role of each individual factor. In addition to clinically driven predictor identification using the input of all stakeholders and the literature, data-driven identification may be used, where computer models may select which predictors to use in in the final model [56]. Another complex aspect of including a predictor is that there may be discussions surrounding the ethics of incorporating specific prognostic factors. For example, the inclusion of factors such as socioeconomic status or parental educational level in the prognosis may seem incorrect or potentially harmful. Parents might perceive this as implying that their child would have had a more favorable outcome if they were wealthier, potentially leading to feelings of guilt.

Second, outcomes in current prognostic models have been dominated by the use of researcher-determined outcome parameters such as survival, mortality, and medical complications including bronchopulmonary dysplasia, retinopathy of prematurity, hydrocephalus, cerebral palsy, and neurodevelopmental impairment [57,58]. More functional outcomes, such as behavioral problems or rehospitalization rates, are less commonly used as outcome measures, for example, due to short follow-up duration or insufficient follow-up programs [5,59,60,61]. These abovenamed researcher-determined outcomes as a metric of the success of NICU treatment do not necessarily always fully align with parental and patient priorities [48,62,63]. The child’s quality of life, ability to communicate, vision and hearing, behavior, or feeding problems are examples of what parents consider as important [64,65], and are as likely to have a significant impact on the daily lives of EPIs and their families [48]. However, not all outcome parameters may be suitable to use as the outcome measures of a prognostic model, nor may they be available as reliable data. As stated previously, NDI is often used as an outcome parameter of long-term follow-up in EPIs and may serve as a suitable outcome parameter for prognostic models. As data on social functioning and mental health are often not available, an alternative option might be to use proxies in a model [18], such as the need for special education or intensive daily support [66]. Thus, choosing the right outcome parameters in a model will involve a combination of finding consensus in relevant outcome parameters among all stakeholders and the availability of reliable data.

Third, prognostic models might inherently be outdated, especially when long-term outcome data are used. Patient populations, neonatal treatments, and outcomes may change over time. Prognostic modeling in a dynamic artificial intelligence environment may be promising, since a continual flow of data may allow for model adjustments [67].

Fourth, it is essential to evaluate the performance (i.e., generalizability) of a clinical prediction model in a population distinct from the one in which it was originally developed. External validation is a critical step in the model development process as it helps assess its general applicability and reliability, thus laying the foundation for informed and shared effective decision making in clinical practice. Subsequently, after external validation, it is essential to assess the model’s impact on clinicians’ behavior and/or clinical outcomes before considering its implementation in clinical practice [68]. There is still much to learn about the actual potential of prognostic models in clinical settings, and the implementation of prognostic models should be studied.

Finally, prognostic models are mainly developed in high-income countries with advanced NICU care [7]. To ensure generalizability, prognostic models should be validated among different countries, healthcare systems, and sociocultural contexts.

## 5. Future Recommendations

Since we did not perform a comprehensive, systematic review, we cannot claim that our recommendations are 100% complete. However, based on our overview above of the opportunities and challenges in the use of prognostic models for EPIs, we make the following four recommendations for the future.

First, the purpose of each prognostic model should be defined, including considering who is going to use the model and in which situation/setting. Specific purposes may be: (I) individual decision making regarding the initiation of a specific treatment decision; (II) the development of a perinatal guideline; and (III) in the process of policy development, such as benchmarking or resource allocation [69].

Second, the purpose of a model must align with the included predictors and the chosen outcome measure. Ideally, all relevant stakeholders, including healthcare professionals and patient representatives, should participate in the selection of parameters, striving to achieve as much consensus as possible regarding the factors to be incorporated in the prognostic model. Recently, a standardized set of 21 core outcomes for neonates covering three domains (physical, social, and mental functioning) was published, after a consensus-driven development process that involved stakeholders and professionals from all over the world [70].

Third, to improve implementation in clinical practice, researchers should explore which outcome definitions are understandable for parents and how to communicate prognostic information to parents. Healthcare providers should be trained on how to discuss the results of prognostic models during counselling [71].

Last, the clinical implications of using a prognostic model for EPIs should be elaborated, and the experiences of both doctors and parents related to this topic have to be evaluated.

## 6. Conclusions

Prognostic models for mortality and severe morbidity may be useful in the context of extremely preterm birth and can be of added value for several stakeholders. Prognostic models may support more uniformity, consistency, and transparency during prenatal counseling conversations. Importantly, the purpose of a prognostic model should be clearly defined, and integrating these models in prenatal counselling requires thoughtful consideration.

## Figures and Tables

**Table 1:** Opportunities (O) and challenges (C) in using prognostic models at population and individual levels.

Using Prognostic Models at a Population Level	
Perinatal guidelines	This will be of added value in the development of perinatal guidelines if we could establish clear-cut thresholds that delineate either acceptable or unacceptable rates of adverse outcomes (O)
Policy development	This could aid in proactive planning for required beds and equipment, by predicting the likelihood of, e.g., length of hospital stay or adverse outcomes (O)
	It could potentially facilitate benchmarking using the ratio between observed and predicted outcomes as a performance indicator to compare NICU outcomes (O)
**Using prognostic models at an individual level**	
Individualizing prenatal counselling and decision making	This would enable the use of a combination of multiple factors to predict the risk of future adverse clinical outcomes in an EPI (O)
	It may increase uniformity in outcome predictions during counselling (O)
	The relevance of a prognostic model in shared decision making regarding resuscitation might be limited (C)
	It is important to consider how the “calculations” from the model are communicated to parents (C)
	It may lead inadvertently to more directive communication and diminish parental involvement in the decision-making process if the meaning of prognostic models is poorly understood (C)

## Data Availability

Data sharing is not applicable. No new data were created or analyzed in this study.
